# Supplementary material for: Getting More Out of Biomedical Documents with GATE's Full Lifecycle Open Source Text Analytics
Source: PLoS Comput Biol. 2013 Feb 7;9(2):e1002854. doi: 10.1371/journal.pcbi.1002854 (PMC3567135; doi:10.1371/journal.pcbi.1002854)
Supplement: Dataset S1 — GATE software. Dataset S1 bundled with this paper contains a distribution of GATE (or see http://gate.ac.uk/download/). (TGZ) [file pcbi.1002854.s001.tgz › plos-gate/doc/18MonthReport.html]

GATE2 18-month report


## GATE 2: 18-month report

### Hamish Cunningham, Valentin Tablan, Cristian Ursu, Kalina Bontcheva, 1st January, 2001

This is the second in a series of progress reports for the GATE 2 project
(EPSRC grant GR/M31699, running from July 1999 to summer 2002).
The previous report is here.
Our starting points are the original
GATE project (GR/K25267 - available at http://www.dcs.shef.ac.uk/nlp/gate/),
and the proposal document for the new project.

Our work over the last 9 months, from April through December
2000 has been to implement the second version of the GATE system
in order to satisfy as many as possible of the requirements identified
in the first phase of the project.

## Use cases

We have identified a number
requirements
for Software
Architecture for Language Engineering (SALE); the version of the system
at half-way through the project meets (partially or completely) the
following subset:

1. **Processing resources**
   1. *Locating, loading and initialising components from local and non-local
      machines.* **[Fully met]**
   2. *Executing processing components, serially or in parallel.*
      **[Partially met]**
   3. *Representing information about components.* **[Fully met]**
   4. *Factoring out commonalities amongst components.*
2. **Language Resources, corpora and annotation**
   1. *Accessing data components.*
   2. *Managing collections of documents (including recordings) and their
      formats.* **[Partially met]**
   3. *Representing information about text and speech.* **[Fully met]**
   4. *Representing information about language.*
   5. *Indexing and retrieving information.* **[Partially met]**
3. **Methods and applications**
   1. *Method support*
      - *FST, unification and statistics over information.* **[Partially
        met]**
      - *Comparing different versions of information.* **[Partially
        met]**
   2. *Application issues*
      - *Storing information.* **[Partially met]**
      - *Deployment and embedding.* **[Fully met]**
   3. *Development issues*
      - *Interoperation with other infrastructures.* **[Partially
        met]**
      - *Viewing and editing data components and information.*
        **[Partially met]**
      - *UI access to architectural facilities (development environments).*
        **[Partially met]**
